# Ladies on Hospital Boards

**Published:** 1901-10-12

**Authors:** 


					LADIES ON HOSPITAL BOARDS.
At the annual meeting of the Koyal Hospital for In-
curables, which will be held in November, an effort will, we
understand, be made to effect an important and much-
needed reform by the introduction of ladies upon the Board
of Management. It has long been felt that in the manage-
ment of a great institution like this, the vast majority of the
inmates of which are of the female sex, women should take
some active part. At the annual dinner held in aid of the
charity in May last the Chairman, Lord Tweedmouth, drew
attention to this point, saying that of the 217 inmates only 36
were males; and the Marquess of Northampton, the President
of the Board, also endorsed the view that the time had come
when ladies should be elected to the Board of Management.
Even so long ago as 1893, after the inquiry held by the Select
Committee of the House of Lords, it was recommended by
the Earl of Aberdeen that ladies should be invited to join
the Board, and at the annual meeting in November 1899 a
memorial, asking that this recommendation should be
carried into effect, signed by Adeline Duchess of Bedford
and many other influential members of the Board, was pre-
sented, but no action was taken. It is high time, however,
that something should be done in the matter, and in view of
the fact that the proposed reform is not only obviously for
the benefit of the institution, but has the support of so many
well-known governors, it is to be hoped that well-wishers of
the institution will take the trouble to attend the annual
meeting and support Miss MaCree, who has consented to
stand for election.

				

